# Editorial for Special Issue “High-Reliability Semiconductor Devices and Integrated Circuits, 3rd Edition”

**DOI:** 10.3390/mi17070841

**Published:** 2026-07-15

**Authors:** Changqing Xu, Yi Liu, Yiqiang Chen

**Affiliations:** 1 Guangzhou Institute of Technology, Xidian University, Guangzhou 510555, China; 2 Faculty of Integrated Circuit, Xidian University, Xi’an 710071, China; yiliu@mail.xidian.edu.cn; 3 Science and Technology on Reliability Physics and Application of Electronic Component Laboratory, The No. 5 Electronics Research Institute of the Ministry of Industry and Information Technology, Guangzhou 510610, China; yiqiang-chen@hotmail.com

Semiconductor devices and integrated circuits are increasingly deployed in automobiles, avionics, aerospace platforms, radiation-monitoring systems, high-power optoelectronics, and other safety- or mission-critical applications. In these fields, reliability is not determined by a single device parameter or failure mechanism. Instead, it depends on the combined influence of material properties, device structures, circuit architectures, packaging technologies, manufacturing variations, environmental stresses, and system operating conditions. Continued transistor scaling and increasing integration density make coupled aging mechanisms and circuit-level reliability modeling increasingly important, while dynamic operation, packaging interactions, ionizing radiation, and extreme environmental loads can further alter device and system performance [1,2,3,4].

Continued device scaling and system integration have therefore increased the complexity of reliability analysis. Radiation exposure may generate defects, modify charge transport and electrical parameters, and ultimately degrade device or circuit functionality [3]. Thermal cycling, mechanical loading, and other extreme conditions can initiate microstructural evolution, interfacial damage, and fatigue in electronic interconnections [4,5]. At the same time, high-performance sensing and readout circuits must maintain wide dynamic range, low noise, fast response, and acceptable power consumption under demanding operating conditions. These coupled effects motivate physics-of-failure analysis, compact and multiscale modeling, and AI-assisted design-for-reliability methods that can accelerate prediction and design optimization [1,5,6].

The Special Issue “High-Reliability Semiconductor Devices and Integrated Circuits” provides a platform for recent advances in device physics, reliability modeling, environmental-effect analysis, circuit design, numerical simulation, and engineering-oriented performance prediction. The eight contributions collected in this Special Issue cover strong-magnetic-field effects, radiation detector degradation, single-event-rate prediction, radiation-aware circuit simulation, packaging fatigue, wide-bandgap ultraviolet detectors, multimodal infrared readout circuits, and high-dynamic-range logarithmic amplifiers. Together, these works illustrate a transition from isolated reliability characterization toward multiscale, application-oriented, and design-integrated reliability research.

Liao et al. (Contribution 1) investigated the electrical behavior of vertical NPN bipolar junction transistors under strong magnetic fields. By analyzing terminal currents, current gain, carrier trajectories, potential distributions, and Hall voltage, the authors showed that magnetic fields modify the carrier distribution and current-transport path within the device. The inherent structural asymmetry of the vertical BJT produces a pronounced magnetic anisotropy, causing the device response to depend strongly on the direction of the applied field. Based on these observations, an interference-resistant structural design was proposed to suppress performance degradation along magnetically sensitive directions. This work provides both physical insight and a device-level optimization strategy for semiconductor components operating in complex magnetic environments.

Zhang et al. (Contribution 2) presented a multimodal CMOS readout integrated circuit for short-wave infrared image sensors. The proposed analog front end combines dual-mode buffered-direct-injection and direct-injection pixels with a column-parallel 12-bit two-step single-slope analog-to-digital converter. The readout circuit dynamically selects the injection mode according to the detector-current condition, balancing the high injection efficiency and bias stability of the buffered structure against the lower power consumption of direct injection. A four-terminal comparator and dynamic reference-voltage compensation are further introduced to mitigate charge leakage and offset. Fabricated in a 0.18 μm CMOS process for a 64 × 64 array, the circuit demonstrates a wide dynamic range, controlled noise, and practical power consumption. The work highlights the importance of adaptive circuit architectures in improving the robustness of sensor interfaces across varying signal and background conditions.

Wang et al. (Contribution 3) developed a high-dynamic-range logarithmic amplifier using a 180 nm SiGe BiCMOS process. A nine-stage fully differential limiting-amplifier chain and a ten-stage rectifier provide a dynamic range exceeding 80 dB over a wide frequency band. The proposed logarithmic-slope adjustment circuit allows the detector response to be configured through an external resistor, accommodating different input-power requirements. A dedicated power-down control mechanism reduces standby power consumption while preserving fast normal-mode response. By jointly considering dynamic range, bandwidth, response time, configurability, and power management, this work offers a practical circuit solution for radar, optical receivers, field-strength meters, and other high-precision electromagnetic measurement systems.

Du et al. (Contribution 4) revisited the prediction of single-event rates in semiconductor circuits. The study analyzes the limitations of conventional rectangular–parallelepiped and integral rectangular–parallelepiped models, particularly when linear energy transfer and measured upset cross-section are used without fully considering particle energy, particle type, target geometry, and radiation–matter interaction mechanisms. The authors derived a set of generalized single-event-rate estimation equations from the probabilistic characteristics of single-event effects and extended the traditional sensitive-volume concept by introducing an interaction volume. The resulting formulation establishes a relationship between the projected single-event area and the event-rate cross-section and can be simplified for engineering applications. Validation on representative circuits demonstrates the potential of the model for more general and physically informed radiation-rate prediction.

Ryzhov et al. (Contribution 5) proposed a radiation-aware corner-analysis methodology for CMOS analog integrated circuits. The approach combines process, voltage, and temperature variations with radiation-induced parameter shifts and is implemented using the open-source QUCS-S and Ngspice environments. A radiation-sensitive field-effect-transistor SPICE macromodel was developed to represent threshold-voltage variation with radiation dose, while percentile-based parameter extraction was used to account for device-to-device and process-corner variations. The authors further proposed a monolithic RADFET radiation sensor integrating the sensing device and CMOS readout circuit on the same die. This work extends conventional PVT analysis into a broader radiation–process–voltage–temperature design framework and demonstrates the growing potential of open-source EDA tools for high-reliability analog design.

Kopyev et al. (Contribution 6) studied the photoresponse and internal-gain behavior of vertical Ni/β-Ga_2_O_3_ Schottky-barrier-diode ultraviolet detectors. The fabricated devices exhibit strong rectification, solar-blind ultraviolet sensitivity, self-powered operation, and increasing responsivity and detectivity under reverse bias. The external quantum efficiency exceeds 100%, indicating significant internal photoconductive gain. Through bias-dependent electrical and temporal characterization, the authors associated this gain with charge trapping and modulation of the effective Schottky barrier. The study clarifies the relationship between electric field, response dynamics, and internal amplification in ultra-wide-bandgap photodetectors, providing useful guidance for the design of sensitive and energy-efficient ultraviolet sensing systems.

Ouyang et al. (Contribution 7) examined the degradation of a p-NiO/β-Ga_2_O_3_ heterojunction X-ray detector after gamma-ray irradiation. The device was characterized under operating bias before and after exposure to a total dose of 13.5 kGy(Si). The results show that trap-assisted conduction affects the pre-irradiation response, while gamma irradiation increases the net carrier concentration and electric field in the β-Ga_2_O_3_, causing Poole–Frenkel emission to dominate the reverse leakage current. These changes reduce detector sensitivity, output linearity, and response speed. The work provides important evidence that even radiation detectors fabricated from nominally radiation-resistant ultra-wide-bandgap materials can undergo substantial performance degradation during long-term operation. It also emphasizes the need to evaluate detector sensitivity and radiation tolerance simultaneously rather than independently.

Cheng et al. (Contribution 8) investigated solder-layer reliability in microchannel-cooled high-power diode lasers for space applications. Using finite-element analysis and the Anand constitutive model, the authors evaluated stress, strain, and fatigue under thermal cycling and random vibration for two packaging configurations. The critical stress–strain regions were found at the solder-layer edges, with failure expected to initiate preferentially along the laser-cavity direction. Random-vibration stresses remained below the solder yield strength, indicating elastic deformation and high-cycle fatigue, whereas thermal cycling produced the dominant accumulated damage. By combining thermal and vibration damage, the study predicted the total fatigue life of both package structures and demonstrated that packaging configuration has a substantial influence on service lifetime. This contribution provides an engineering basis for reliability-oriented packaging optimization of semiconductor lasers in space environments.

Taken together, these contributions reveal several important directions in high-reliability semiconductor research.

First, reliability analysis is moving beyond conventional electrical-stress evaluation toward a broader treatment of special environments. Strong magnetic fields, ionizing radiation, thermal cycling, vibration, and high-energy particle interactions influence different physical layers of electronic systems. Their effects may begin with changes in carrier transport or defect states but can ultimately alter circuit accuracy, detector sensitivity, package lifetime, or system-level availability. Establishing the links between environmental conditions and functional performance is therefore essential.

Second, wide-bandgap and ultra-wide-bandgap semiconductors are becoming increasingly important for high-reliability sensing and power applications. The two β-Ga_2_O_3_-based detector studies demonstrate both the considerable advantages and the unresolved reliability challenges of these materials. High responsivity, solar-blind operation, low dark current, and self-powered detection can coexist with trap-controlled conduction, bias-dependent gain, and cumulative radiation degradation. Future work should place greater emphasis on the interaction among material defects, interface states, electric-field distribution, and long-term environmental exposure.

Third, reliability must be incorporated into circuit and system design rather than evaluated only after implementation. The multimodal infrared readout circuit adapts its operating mode to changing detector conditions, while the logarithmic amplifier combines signal-range expansion with adjustable slope and standby-power control. The radiation-aware corner-analysis approach similarly treats radiation dose as an additional design dimension alongside process, voltage, and temperature. These examples illustrate that adaptive operation, calibration, and environment-aware simulation are increasingly important elements of reliable integrated-circuit design.

Fourth, physically grounded prediction and high-efficiency simulation remain central research needs. Generalized single-event-rate models are required as device geometries and radiation-interaction mechanisms become more complex. At the circuit level, reliability evaluation should account for statistical process variation and environmental degradation simultaneously. At the package level, fatigue models must connect localized stress–strain distributions to realistic mission profiles. Combining physical models, experimental data, statistical methods, and efficient numerical tools will be crucial for reducing evaluation cost without sacrificing credibility.

Finally, high-reliability design is inherently multiscale. Device geometry determines carrier transport in magnetic fields; defects and interfaces govern photoconductive gain and radiation degradation; circuit architectures determine noise, dynamic range, and power consumption; and packaging structures govern mechanical and thermal lifetime. Future reliability platforms should therefore support coordinated analysis across materials, devices, circuits, packages, and systems.

Further progress is expected from multiphysics digital twins, radiation-aware process design kits, uncertainty-quantified compact models, automated reliability optimization, and artificial-intelligence-assisted design-space exploration. Data-driven methods may improve parameter extraction, degradation prediction, and adaptive control, but their reliability will depend on the quality and representativeness of the underlying physical and experimental data. The most promising direction is therefore not to replace physical analysis with intelligent methods, but to integrate physical knowledge, numerical simulation, experimental validation, and machine learning into a unified reliability-design framework.

The studies presented in this Special Issue demonstrate the diversity and vitality of current research on high-reliability semiconductor devices and integrated circuits. They also show that reliable electronic systems require coordinated advances in materials, device structures, circuit techniques, environmental modeling, and packaging design. We hope that these contributions will stimulate further interdisciplinary research and support the development of more robust semiconductor technologies for automobiles, aerospace systems, radiation environments, sensing platforms, and other demanding applications.

We sincerely thank all authors for their valuable contributions to this Special Issue. We also thank the reviewers for their careful evaluations and constructive suggestions, which helped improve the scientific quality of the published papers. Finally, we acknowledge the editorial team of *Micromachines* for their professional assistance and efficient management throughout the preparation and publication of this Special Issue.
